# Can we see what is invisible? The role of MRI in the evaluation and management of patients with pathological nipple discharge

**DOI:** 10.1007/s10549-019-05321-w

**Published:** 2019-07-27

**Authors:** Konstantinos Zacharioudakis, Theodoros Kontoulis, John X. Vella, Jade Zhao, Rathi Ramakrishnan, Deborah A. Cunningham, Ragheed Al Mufti, Daniel Richard Leff, Paul Thiruchelvam, Katy Hogben, Dimitri J. Hadjiminas

**Affiliations:** 1 Breast Unit, Charring Cross Hospital, Imperial College Healthcare NHS Trust, Fulham Palace Rd, Hammersmith, London, W6 8RF UK; 2 Breast Unit, Nightingale Centre, Wythenshawe Hospital, Manchester University NHS Foundation Trust, Southmoor Road, Wythenshawe, Manchester, M23 9LT UK; 3 Department of Surgery and Cancer Imperial College London, Ayrton Rd, Kensington, London, SW7 5NH UK

**Keywords:** Breast cancer, Nipple discharge, MRI, DCIS, Occult breast cancer

## Abstract

**Introduction:**

The aim of this study was to determine the ability of MRI to identify and assess the extent of disease in patients with pathological nipple discharge (PND) with an occult malignancy not evident on standard pre-operative evaluation with mammography and ultrasound.

**Methods:**

Patients presenting to the breast unit of Imperial College Healthcare NHS Trust between December 2009 and December 2018 with PND and normal imaging were enrolled in the study. Pre-operative bilateral breast MRI was performed in all patients as part of our protocol and all patients were offered diagnostic microdochectomy.

**Results:**

A total of 82 patients fulfilled our selection criteria and were enrolled in our study. The presence of an intraductal papilloma (IDP) was identified as the cause of PND in 38 patients (46.3%), 14 patients had duct ectasia (DE-17%) and 5 patients had both an IDP and DE. Other benign causes were identified in 11 patients (13.4%). Despite normal mammography and ultrasound a malignancy was identified in 14 patients (17%). Eleven patients had DCIS (13.4%), two had invasive lobular carcinoma and one patient had an invasive ductal carcinoma. The sensitivity of MRI in detecting an occult malignancy was 85.71% and the specificity was 98.53%. The positive predictive value was 92.31% and the negative predictive value was 97.1%.

**Conclusions:**

Although a negative MRI does not exclude the presence of an occult malignancy the high sensitivity and specificity of this diagnostic modality can guide the surgeon and alter the management of patients with PND.

## Introduction

Nipple discharge accounts for 5–12% of referrals to a breast clinic and is the second most common indication for a breast procedure [1, 2]. Pathologic nipple discharge (PND) is defined as unilateral single duct nipple discharge that is persistent and spontaneous in a non-lactating woman. Although the most common causes for PND are benign in 10–15% of cases PND is caused by the presence of an underlying malignancy, usually DCIS [3].

The initial diagnostic workup for a patient presenting with PND includes a detailed history, clinical breast examination, breast imaging and smear cytology. Any suspicious finding from clinical breast examination (CBE) or imaging then follows the normal pathway of triple assessment.

Although there is a higher incidence of breast cancer in patients with bloodstained nipple discharge, colour is not an outright determinant of diagnosis since an underlying malignancy can be present with any colour of PND [4]. Smear cytology has a low sensitivity (26.7%) in the evaluation of a patient with PND for malignancy especially in the absence of blood with a false positive rate of 2.7% and a false negative rate of 20–35% [1]. Despite its inherent flaws, the presence of epithelial cells or red blood cells on smear cytology of patients with PND and normal imaging identifies a group of patients with a 10% chance of malignancy and is therefore considered an important diagnostic step in our unit [5].

Although minimally invasive diagnostic methods such as ductal aspiration (lavage), galactography (ductography) and ductoscopy have been used to evaluate PND, they have not become the standard of care due to their limitations. Therefore, in the majority of cases where a precise pre-operative diagnosis is not possible due to normal imaging, microdochectomy is performed for diagnostic purposes and symptomatic relief. Although histological examination of the microdochectomy specimen confirms the presence of malignancy, its extent is obscure given the normal pre-operative imaging making definitive surgical management problematic.

There is increasing evidence in the literature showing that breast MRI allows the diagnosis of DCIS in cases that could go undetected by mammography [6]. In fact it is suggested that MRI detects a different subset of DCIS that does not exhibit microcalcifications and therefore remains mammografically occult [7].

The aim of this study was to determine the ability of MRI to identify and assess the extent of disease in patients with PND with an occult malignancy not evident on standard pre-operative evaluation with mammography and ultrasound.

### Patients and methods

Patients presenting to the breast unit of Imperial College Healthcare NHS Trust between December 2009 and December 2018 with spontaneous single duct nipple discharge containing epithelial cells or blood and normal mammogram and ultrasound were enrolled prospectively in our study. All patients underwent triple assessment i.e. CBE, breast ultrasound, bilateral mammography and nipple discharge cytology. Women with multiple duct discharge or positive findings from CBE or imaging were not included in this study. Patients unable to undergo MRI because of claustrophobia were also excluded.

In accordance with our institutions’ protocol all women with epithelial cells or blood present in their nipple smear and normal CBE and imaging were offered microdochectomy for diagnostic purposes. Pre-operative bilateral breast MRI was performed in all patients as part of the study. The results of the MRI did not affect the patients’ management i.e. a positive finding did not alter our decision to proceed with the microdochectomy. Patients that had epithelial cells in their smears but declined surgery or were deemed high risk for anaesthesia were excluded from our study. Patients with no cells present in their nipple smear did not have an MRI and were followed up with repeated annual CBE, conventional imaging and nipple smear cytology for 3 years or until their symptoms resolved.

In total, 73 patients underwent microdochectomy under general anaesthesia according to a previously described technique [5]. There were nine patients who underwent major duct excision (Hadfields operation) due to inability to identify the involved duct on the day of the operation. Patients fulfilling all other inclusion criteria who underwent major duct excision were also included in our study. Patients were discharged from hospital on the same day.

Images were obtained either using GE Signa advantage 1.5T or 3T scanners, or Siemens Avanto 1.5T or Siemens Verio 3T scanners using dedicated breast coils. Contrast enhanced scans were obtained after an intravenous injection of 0.2 mls/kg of Gadoteridol (ProHance) at 3 ml/sec followed by a saline flush of 20cc. Subtraction images and multiplanar reconstructions were derived from the dynamic study data-set. All images were interpreted by an experienced consultant Breast Radiologist before surgery and were therefore blind to the final diagnosis.

The imaging diagnoses were dichotomised as follows: diagnoses coded as BI-RADS 1,2,3 were deemed to be negative, BI-RADS categories 4 and 5 were deemed to be positive.

MRI findings were not usually followed by MRI-guided biopsies because we have found that these procedures interfere significantly with the success of microdochectomy, which is often rendered impossible after an MRI-guided vacuum-assisted biopsy. Furthermore, the mere presence of a papilloma or other MRI-detected benign lesion does not exclude malignant pathology as the actual cause of SDND, hence in this cohort of consecutive patients and until we are satisfied that the positive predictive value of MRI is sufficiently high, we typically performed microdochectomy after the MRI for histological diagnosis of the cause of SDND. In some cases, a second-look, ultrasound-guided 18G core biopsy was taken from a potential papilloma and as our confidence in MRI increased in the last two cases an MRI-guided biopsy was performed to confirm cancer.

## Results

**Fig. 1:**
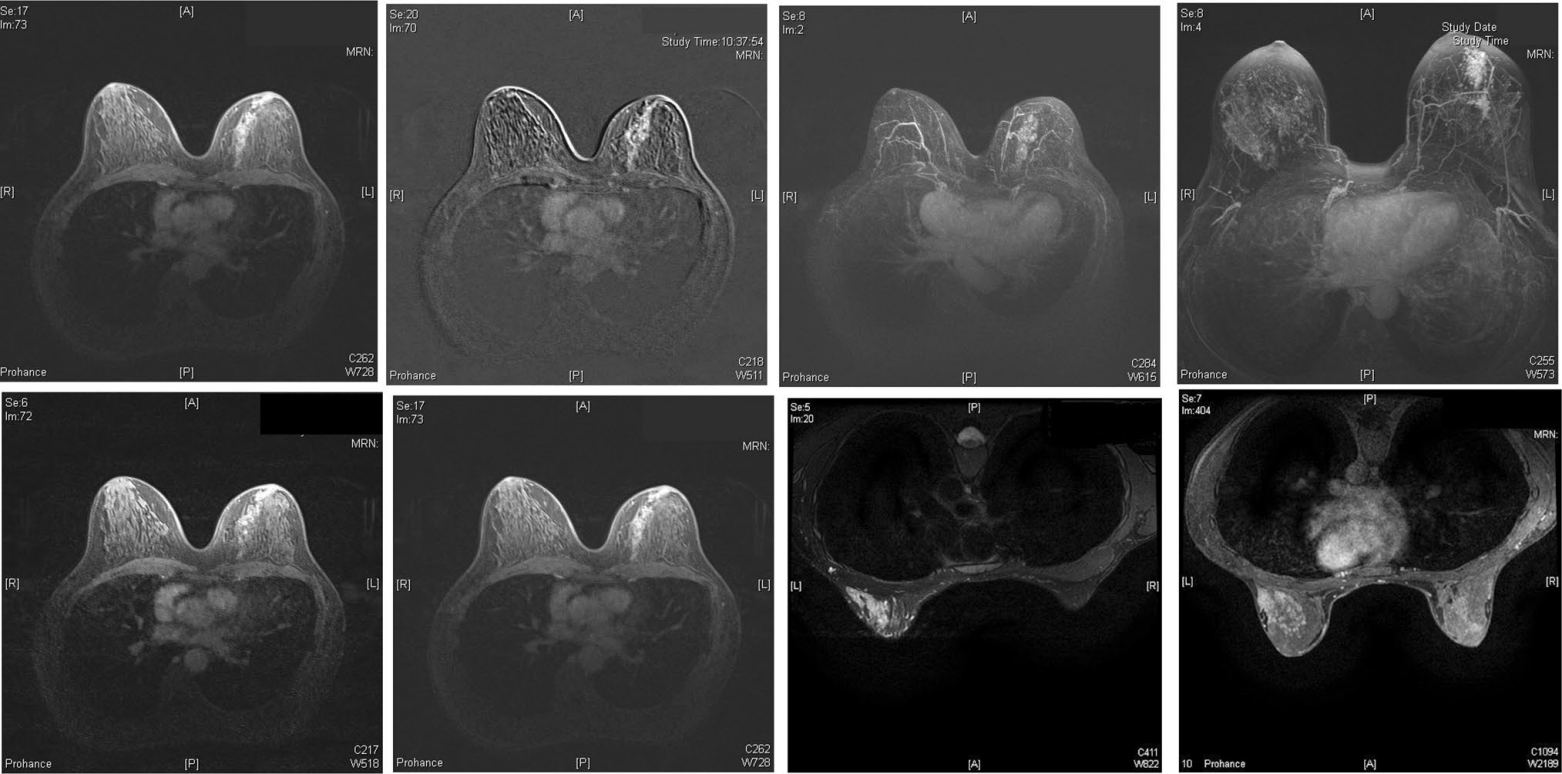
Breast MRIs demonstrating enhancement with ductal/segmental distribution consistent with DCIS in four patients with PND and normal mammography and breast ultrasound

A total of 82 patients fulfilled our selection criteria and were enrolled in our study. Patients with a benign underlying cause were younger than those with a malignant lesion (47.33 vs. 53.62 years *p* = 0.126). The presence of an intraductal papilloma (IDP) was identified as the cause of SDND in 38 patients (46.3%), 14 patients had duct ectasia (DE-17%) and five patients had both an IDP and DE (6%). Other benign causes were identified in 11 patients (13.4%). Despite normal mammography and ultrasound scan, a malignant lesion was identified in 14 patients (17%) in our cohort. Eleven out of these fourteen patients had DCIS on the microdochectomy specimen. Two patients had invasive lobular carcinoma with adjacent DCIS and one patient had a benign papilloma on microdochectomy but the MRI showed an area with grade 2 invasive ductal carcinoma and low grade DCIS in the ipsilateral breast (Table 1). All cases that were correctly diagnosed by MRI as DCIS were characterised by a segmental non-mass suspicious enhancement (Fig. 1).

**Table 1:** Causes of pathologic nipple discharge in patients with normal conventional imaging

Cause	N	%
Total	82	
Intraductal papiloma (IDP)	38	46.3
Duct ectasia (DE)	14	17
IDP + DE	5	6
Other benign	11	13.4
Malignancy	14	17.07
DCIS	11	13.41
ILC	2	2.4
IDC	1	1.2

Ten out of the fourteen patients with a malignant histology had a mastectomy and sentinel lymph node biopsy as the final operation. One patient had re-excision of margins and three patients had wire guided WLE. Pre-operative breast MRI identified the presence of cancer in 12 out of the 14 patients. The sensitivity of MRI in detecting an occult malignancy was 85.71% (95% CI 57.19%–98.22%) and the specificity was 98.53% (95% CI 92.08%–99.96%). The positive predictive value was 92.31% (95% CI 62.89%–98.84%) and the negative predictive value 97.10% (95% CI 90.27%–99.18%) (Table 2).

**Table 2:** Diagnostic accuracy of MRI in patients presenting with PND, normal mammography and breast ultrasound and an underlying malignancy

Statistic	Value	95% CI
Sensitivity	85.71%	57.19–98.22%
Specificity	98.53%	92.08–99.96%
Positive likelihood ratio	58.29	8.23–412.65
Negative likelihood ratio	0.14	0.04–0.52
Disease prevalence	17.07%	9.66–26.98%
Positive predictive value	92.31%	62.89–98.84%
Negative predictive value	97.10%	90.27–99.18%

## Discussion

Although the presence of a mammographic or sonographic finding in a patient presenting with SDND significantly increases the risk of breast cancer [8] most patients with PND have normal imaging [9]. Based on our protocol, only patients with PND, normal imaging (mammography, ultrasonography) and epithelial cells or blood present in their PND were included in our study. The incidence of breast cancer (in situ or invasive) was 17% (14/82) with the majority of these patients (11/14) having DCIS. Our findings are concordant with the literature. In a cohort of patients reported by Morrogh et al. with PND and normal ultrasound and mammography the incidence of breast cancer was 10% [9]. Fisher et al. reported similar results in a series of 121 patients with normal breast imaging and PND with a diagnosis of DCIS in 7% of patients [10]. However, as both studies were retrospective and included patients with acellular smears the incidence of breast cancer might have been diluted in their results. In our prospective cohort only patients with the presence of epithelial cells or red blood cells in their nipple discharge were included which could explain the higher incidence of invasive or in situ carcinoma.

Variable diagnostic and therapeutic approaches have been proposed in order to further evaluate PND such as galactography or ductal aspiration (lavage) but have not become standard medical practice [2]. Fiberoptic ductoscopy is a promising alternative that allows direct visualisation of the ductal system [10]. It is however an expensive and time consuming procedure that requires advanced skills and has limitations in its use, especially for lesions located distally. Consequently, its role in breast cancer management remains limited outside clinical trials.

As a result, surgery still remains the gold standard for diagnosis and treatment of SDND providing a definitive histological diagnosis while at the same time alleviating the patient from a troublesome symptom [11]. The two most commonly performed operations are microdochectomy and radical sub-areolar duct excision (Hadfield’s operation). It is our opinion that provided the affected duct is correctly identified and isolated microdochectomy is superior to Hadfield’s operation. Microdochectomy does not cause major interference to the rest of the breast and is associated with minimal morbidity. Furthermore, up to 20% of lesions causing SDND are located distal to the area included in a sub-areolar duct excision but are usually included in the microdochectomy specimen [1]. The use of methylene blue allows the surgeon to identify distal side branches thus increasing the likelihood of finding the cause of SDND [1, 12].

In cases where the presence and extent of disease is unknown from conventional imaging and the diagnosis of in situ or invasive cancer is made following microdochectomy we are faced with a dilemma regarding further surgical management i.e. whether to proceed with a mastectomy or breast conserving surgery.

In our series pre-operative breast MRI outlined the extent of pathology in 12 out of the 14 patients with an underlying malignancy, thus allowing us to plan further surgical management once the diagnosis was confirmed. All 12 patients with a true positive MRI presented with segmental non-mass enhancement. Towards the end of the study period we were persuaded that this particular pattern of segmental non-mass enhancement on MRI is always malignant, hence we confirmed the diagnosis in the last two cancers by MRI-guided biopsy. There were two patients with DCIS on the microdochectomy specimen and a false negative MRI. In the first case the extent of the disease was minimal (2 mm) and the patient underwent re-excision of margins that was negative therefore it is unsurprising that the DCIS was not visualised on MRI. The second patient had extensive disease occupying most of her breast and underwent mastectomy. She presented 6 months later with extensive DCIS on the other breast that required contralateral mastectomy. One could speculate that as she most likely had synchronous bilateral mammographically occult DCIS at her initial presentation the bilateral non-mass enhancement on MRI was erroneously classified as benign.

In our cohort the sensitivity of MRI for malignant lesions was 85.71% (95% CI 57.19% to 98.22%) and the specificity was 98.55% (95% CI 92.19% to 99.96%). The high NPV (97.1%–95% CI 90.27% to 99.18%) of MRI as a diagnostic study could suggest that a negative study might obviate the need for a microdochectomy in a subgroup of patients with favourable clinical characteristics. Furthermore, in patients with a positive finding from MRI, a second-look ultrasound or an MRI-guided needle core biopsy can precede any surgical intervention. The surgeon can then plan the extent and type of surgery based on the MRI.

Orel et al. were among the first to investigate the use of breast MRI in patients with nipple discharge and normal mammogram in a cohort of 23 patients of whom 15 underwent subsequent excision biopsy [13]. MRI findings were suspicious in six of the seven patients with an underlying malignancy. In a study by Lubina et al. [14] MRI was compared with galactography in 50 patients with nipple discharge and a negative mammogram and ultrasound scan. A malignant lesion was present in 14.8% of the patients and the sensitivity of MRI in detecting breast cancer was 75% (95% CI 34.91% to 96.81%) while the specificity was 75% (95% CI 60.40% to 86.36%). In a meta-analysis by Berger et al. the pooled sensitivity and specificity of MRI for cancer detection in patients with pathological nipple discharge was 92% (95% CI, 74–98%) and 97% (95% CI, 80–100%), respectively [15]. However, in the latter meta-analysis 8 out of the 10 studies that were included were retrospective and in the 2 prospective studies the MRI was not compared to microdochectomy as a gold standard. Our prospective series included 82 consecutive patients presenting in a single breast unit over a period of 9 years with PND and negative conventional imaging. Regardless of the MRI findings all patients underwent diagnostic surgical excision (either microdochectomy or major duct excision). This robust methodology strengthens the validity of our findings.

Compared to mammography MRI has a higher sensitivity for the detection of breast cancer and is not affected by breast density [16]. In a systematic review published by Warner et al. on the use of magnetic resonance imaging to screen women at high risk for breast cancer the sensitivity of mammography was 32% (95% CI 62–88) whereas the sensitivity of MRI as a screening tool in this high risk population was 75%(95% CI 62–88) [6]. When the two procedures where combined the sensitivity was increased to 84% (95% CI 70–97). The positive predictive value was 87.50% (95% CI 47.35%–99.68%) and the negative predictive value 94.55% (95% CI 84.88%–98.86).

Although in all studies included in the latter systematic review MRIs’ sensitivity in detecting invasive cancer was superior to mammography, the results were not clear regarding the sensitivity of MRI in detecting ductal carcinoma in situ (DCIS). Kuhl et al. published the results of a prospective observational study on the use of MRI for the diagnosis of pure DCIS in women with a family history of breast cancer and a lifetime risk of 20% or more based on the geneticists’ assessment showing that the sensitivity of MRI in detecting DCIS in this cohort far surpassed that of mammography [7]. In this study, 43% of intraductal carcinomas were mammographically occult and were diagnosed on MRI alone. The results of this study suggested that the sensitivity of mammography for diagnosis of DCIS is limited. We can only speculate on the diagnostic and prognostic implications of these mammographically occult lesions.

The diagnosis of breast disease on MRI is based on tissue contrast material enhancement which depends on increased microvessel density or permeability [17]. In our series MRI identified a different subset of DCIS in patients presenting with SDND that did not exhibit microcalcifications and therefore had remained mammograhically occult. One could speculate that in this subgroup of patients DCIS is diagnosed prior to the development of microcalcifications due to the presence of PND and that the same pathophysiologic changes that result in nipple discharge allow its identification through a different modality with the use of MRI.

## Conclusion

Our results support the use of MRI as part of the diagnostic workup of patients presenting with PND and normal mammography and breast ultrasound. Although a negative MRI does not exclude the presence of an occult malignancy the high sensitivity and specificity of this diagnostic modality can alter the management of patients with an underlying malignancy and can guide the surgeon on the extent and type of surgery that is required.

## Abbreviations

PND: Pathologic nipple discharge
DCIS: Ductal carcinoma in situ
IDC: Invasive ductal carcinoma
ILC: Invasive lobular carcinoma
IDP: Intraductal papiloma
DE: Duct ectasia
SDND: Single duct nipple discharge
